# Identification and Antioxidant Activity of Flavonoids Extracted from Xinjiang Jujube (*Ziziphus jujube Mill*.) Leaves with Ultra-High Pressure Extraction Technology

**DOI:** 10.3390/molecules24010122

**Published:** 2018-12-30

**Authors:** Li Zhang, Pengzhan Liu, Linlin Li, Ying Huang, Yunfeng Pu, Xujie Hou, Lijun Song

**Affiliations:** 1 College of Life Science, Tarim University, Alar 843300, China; cxbh1984@163.com (L.Z.); huangying880712@sina.com (Y.H.); yfpu@zju.edu.cn (Y.P.); houxujie@sina.com (X.H.); 2 Xinjiang Product & Construction Group Key Laboratory of Agricultural Products Processing in Xinjiang South, Alar 843300, China; 3 School of Food Science and Engineering, South China University of Technology, Guangzhou 510641, China; lpzhan@scut.edu.cn (P.L.); linlinlee1001@163.com (L.L.)

**Keywords:** ultra-high pressure extraction, UPLC-ESI-MS, Xinjiang jujube leaf, flavonoid, antioxidant activity

## Abstract

In this study, the ultra-high pressure extraction (UHPE) conditions for obtaining the maximum flavonoid yield from Xinjiang jujube (*Ziziphus jujuba Mill*.) leaves (XJL) were optimized by response surface methodology (RSM). Box–Behnken design (BBD) was applied to evaluate the effects of four variables (extraction temperature, pressure, time and liquid-to-solid ratio) on flavonoid yield. The results showed that the optimal flavonoid yield (25.45 ± 0.21 mg/g) was derived at 50.0 °C, 342.39 MPa, 11.56 min, and a liquid-to-solid ratio of 43.95 mL/g. Eight compounds were tentatively identified and quantified as kaempferol and quercetin glycosides with UPLC-ESI-MS. Compared to ultrasound-assisted extraction (UAE), UHPE can obtain higher concentrations of total flavonoids and stronger DPPH and ABTS radical-scavenging activities in a much shorter time. Therefore, UHPE is an alternative to UAE for obtaining flavonoids from XJL, which may be an optional method for large-scale industrial flavonoid extraction from XJL.

## 1. Introduction 

*Ziziphus jujuba Mill*., a plant belonging to the family *Rhamnaceae*, is an important economic crop in East Asia. Chinese jujube can be traced back 4000 years, with over 700 subspecies used during thousands of years of cultivation in the temperate and subtropical areas of the Northern Hemisphere [1]. Jujube is an important fruit in China and has attracted significant interest because of its common consumption as food, a food additive, as a flavoring and in traditional Chinese medicine (TCM) [1,2]. 

The jujube leaf, which is the main byproduct of jujube, has also been used in TCM for thousands of years (i.e., to improve sleep, to nourish the heart and soothe the nerves, and to reduce hemorrhaging and diarrhea) [3,4,5,6]. Modern studies found that the jujube leaves were rich in bioactive components and have various physiological and pharmacological functions [5,6]. For example, the aqueous ethanol extracts of the jujube leaf were used as energetic constituent for hepatosis and wound healing in animal trials [7,8]. Jujube-leaf green tea extracts can inhibit human hepatocellular carcinoma cells via activating AMP-activated protein kinase (AMPK) [9], and an unidentified β-d-glucosidase inhibitor also exists in jujube leaf extract [10]. 

Flavonoids were believed to be the major bioactive components in jujube leaves, which have been shown to be responsible for cardioprotective, anticancer, antidiabetic, anti-aging and neuroprotective effects [3,4,11,12]. Most studies focused on the bioactivity and chemical constituents of jujube seeds and fruits, and less attention was devoted to the jujube leaf. Only two papers have reported flavonoids in methanol extracts of jujube leaf [13,14]. However, the screening and identification of the flavonoids in XJL is still needed.

The number of bioactive compounds recovered from plant materials depends on the extraction methods used. Many approaches are already under active investigation, including solvent extraction (SE), microwave-assisted extraction (MAE), supercritical fluid extraction (SFE), ultrasound-assisted extraction (UAE) and many others [15]. SE for flavonoids from plant materials usually needs a long extraction time, which can cause the loss of flavonoids due to ionization, hydrolysis and oxidation [16]. MAE can penetrate the plant matrix and generate heat within the cells, which also results in cell rupture and mass transfer intensification, but this runs the risk of the severe thermal degradation of flavonoids [17]. SFE is recognized as an ideal method to extract bioactive compounds because it is nontoxic, nonexplosive, and easy to remove from the final extract. However, the cost of SFE is higher than that of most other extraction methods [18]. UAE is widely used because of its capillary effects and enhancing influence on mass transfer [19]. 

Ultra-high pressure extraction (UHPE) operates under high pressures, which can not only destroy the cell wall, cell membrane and other structures of plant materials, but also significantly affect the diffusion and solubility of the target compounds in solvents. Therefore, UHPE has many advantages, such as shortening the time, reducing the temperature, and lowering the solvent and energy consumption compared to the above methods [20]. 

To date, UHPE has never been used in the extraction of flavonoids from XJL. Therefore, this work aimed to investigate the potential of UHPE in XJL flavonoid extraction. The effect of extraction temperature, time, pressure and the liquid-to-solid ratio on the performance of UHPE processes was investigated. The flavonoid yield and antioxidant activity were determined. Particularly, the extracts were also analyzed by UPLC-ESI-MS to identify and quantify major flavonoid profiles. The results will provide a better understanding and identification of the antioxidant compounds of XJL for further investigation and development into value-added foods and neutraceuticals. 

## 2. Materials and Methods

### 2.1. XJL Sample

XJLs (*Ziziphus jujuba Mill. cv. Junzao*) were hand-picked from the Germplasm Resources Base of Tarim University, Alaer City (Latitude: 44°55′N, Longitude: 81°28′E, Xinjiang Province, China) in October 2017. Leaves without disease or mechanical injury were randomly collected from the tree. After collecting, the samples were cleaned and dried in a lyophilizer (ALPHA1-2 LD plus, Christ Alpha, Germany). All dried samples were ground, sifted through a 60-mesh sieve and stored at −18 °C for further analysis.

### 2.2. Chemicals and Reagents

HPLC-grade hyperoside, kaempferol-3-*O*-rutinoside, rutin, quercetin-3-*O*-d-glucosyl-(1→2)-rhamnoside, 2,2-diphenyl-1-picrylhydrazyl (DPPH) and 2,2′-azino-bis(3-ethylbenzothiazoline-6-sulphonic acid) (ABTS) were purchased from Sigma (St. Louis, MO, USA). HPLC-grade methanol, acetonitrile and formic acid were purchased from J&K Scientific Ltd. (Shanghai, China). The water used for HPLC was purified with a Milli-Q Plus system (Millipore, Billerica, MA, USA). All other chemicals were of analytical grade and purchased from Chengdu Chemical Industry (Chengdu, China).

### 2.3. Ultra-High Pressure Extraction (UHPE) 

The UHPE process was performed using an ultra-high pressure system (HPP.L.2-600/0.6, Huatai Senmiao Bioengineering Technology Co., Ltd., Tianjin, China) [20]. Briefly, 5 g of dried XJL powdered sample was accurately weighted and mixed with 70% methanol, then each mixture was vacuum packed and extracted at different operational conditions, including temperature, pressure, time and sample-to-solvent ratio. The pressure and temperature-transmitting media was water, and the extraction process was auto-controlled by computer software. After depressurization, the extracted solutions were separately collected, then filtered and stored at −20 °C.

### 2.4. Ultrasound-Assisted Extraction (UAE)

A sample (5 g) was mixed with 70% methanol in a beaker. After mixing, the beaker was placed in an ultrasonic extractor (XY-2008, Xiyu Instruments Co., Ltd., Shanghai, China) at 20 kHz and 750 W for 1.5 h at 30 °C. After extraction, the extracts were centrifuged, evaporated and stored at −20 °C for further analysis.

### 2.5. Determination of Total Flavonoid Content (TFC)

The method described by Xi et al. [21] was used to determine TFC. Briefly, 0.5 mL of sample was transferred to a 10 mL colorimetric tube and mixed with 0.5 mL of 5% (*w*/*v*) sodium nitrite, 0.5 mL of 10% (*w*/*v*) aluminum chloride and 4 mL of 4% (*w*/*v*) sodium hydroxide thoroughly. The final volume of the mixture was adjusted to 10 mL with 95% (*v*/*v*) ethanol. 

Then, the mixture was incubated for 15 min at room temperature. The absorbance at 500 nm was determined against the distilled water blank solution by a UV-vis spectrophotometer (TU-1901, Purkinje General Instrument Co., Ltd., Beijing, China). The number of total flavonoids was expressed as rutin equivalents through the standard calibration curve (*y* = 0.1126*x* + 161, *R*^2^ = 0.9967). The total flavonoid yield was measured using the following equation:

(1) $$\mathrm{Yield}\;\left(\mathrm{mg}/g\right)=\frac{\mathrm{the}\;\mathrm{mass}\;\mathrm{of}\;\mathrm{extracted}\;\mathrm{flavonoids}\;\left(\mathrm{mg}\right)}{\mathrm{the}\;\mathrm{mass}\;\mathrm{of}\;\mathrm{dried}\;\mathrm{sample}\;\left(g\right)}$$

### 2.6. HPLC Analysis

HPLC analysis was performed on a Thermo Fisher system with an ultraviolet detector and Chameleon 7 software. Separation was achieved by a reversed-phase Eclipse XDB-C18 column (250 × 4.6 mm, 5 μm, Agilent, Santa Clara, CA, USA). The mobile phase consisted of acetonitrile (A) and 1% aqueous acetic acid solution (B). Samples were eluted with the following gradient: 5% A from the start, then up to 20% A at 25 min, from 20 to 50% A in 15 min, from 50 to 90% in 5 min, and from 90 to 5% A in the last 5 min to re-establish the initial conditions before the injection of another sample. All gradients were linear. The flow rate was 1 mL/min, and the injection volume was 10 μL. The column temperature was maintained at 25 °C.

Each compound was identified by its retention time and spiked with standards under the same conditions. The identities were confirmed by UHPLC-MS with ultraviolet spectra. Some compounds were quantified according to the peak area measurements, which were reported in the corresponding standards. The compounds without corresponding standards were quantified by calibration curves of those standards that had similar chemical structures of their isomers.

### 2.7. UPLC-ESI-MS Spectrometry Conditions

Samples were subjected to mass spectrometric analysis using an Orbitrap Fusion Tribrid (Thermo Fisher Scientific) equipped with a Dionex Ultimate 3000 HPLC system (Thermo Fisher Scientific) operating in heated ESI mode.

A reversed-phase Kinetex C18 column (100 × 2.1 mm, 2.6 μm, Phenomenex Corporation, CA, USA) was employed for the UPLC separation. The mobile phases consisted of 1% formic acid solution (solution A) and acetonitrile (solution B). The gradient elution was conducted by adjusting the volume ratio of solution A in the mobile phase: 0–0.1 min, 95% A; 0.1–20 min, 95–70% A; 20–30 min, 70–10% A; 30–35 min, 10% A; 35–36 min, 10–95% A; and 36–40 min, 95% A, at a flow rate of 0.5 mL/min. The injection volume was 10 μL. Mass spectra in positive-ion or negative-ion mode were recorded within 40 min. The spray voltage in the positive ion was 3.5 KV, while it was 3.0 KV in negative ion. The ion transfer tube temperature was 320 °C and the vaporizer temperature was 350 °C. The scan range was 100–1000 *m*/*z*.

### 2.8. Determination of Antioxidant Activities

The XJL flavonoids were extracted according to the optimized extraction process (50.0 °C, 342.39 MPa, 11.56 min, and a liquid-to-solid ratio of 43.95 mL/g). The crude extract was centrifuged at 5000 rmp for 5 min, the supernatants were concentrated by rotary evaporation and the total flavonoid was determined. The extract was then diluted to a solution of different total flavonoid concentrations for further antioxidant activity analysis. 

The DPPH radical-scavenging activity was carried out according to the method of Sarikurkcu et al. [22] with minor modifications. Briefly, 1.0 mL of sample solution with different concentrations (0.1, 0.2, 0.3, 0.4, 0.5 and 0.6 mg/mL) was added to a 4 mL of 0.004% methanol solution of DPPH. The absorbance was read at 517 nm after 30 min incubation at room temperature in the dark. Ascorbic acid was used as a standard.

The DPPH radical-scavenging activity was calculated according to the following equation:

(2) $$\mathrm{DPPH}\;\mathrm{scavenging}\;\mathrm{activity}\left(\%\right)=\left(1-\frac{A_{i}-A_{j}}{A_{c}}\right)\times 100\%$$

where *A_c_* was the absorbance of DPPH solution without sample (2 mL DPPH + 2 mL of 95% ethanol); *A_i_* was the absorbance of the test sample mixed with DPPH solution (2 mL sample + 2 mL DPPH) and *A_j_* was the absorbance of the sample without DPPH solution (2 mL sample + 2 mL of 95% ethanol).

The ABTS radical-scavenging activity was carried out based on the method of Gan and Latiff with some modifications [23]. Briefly, ABTS^+^ was produced directly by reacting 7 mM ABTS solution with 2.45 mM potassium persulfate and allowing the mixture to stand for 16 h at room temperature in the dark. Prior to beginning the assay, the ABTS solution was diluted with methanol to obtain an absorbance of 0.700 ± 0.02 at 734 nm. One milliliter of sample solution with different concentrations (0.1, 0.2, 0.3, 0.4, 0.5 and 0.6 mg/mL) was added to the ABTS solution (2 mL) and mixed. The sample absorbance was read at 734 nm after 30 min incubation at room temperature. Ascorbic acid was used as a standard. The ABTS radical-scavenging activity was calculated according to the following equation:

(3) $${\mathrm{ABTS}}^{+}\;\mathrm{scavenging}\;\mathrm{activity}\left(\%\right)=\left(1-\frac{A_{2}-A_{1}}{A_{0}}\right)\times 100\%$$

where *A*_0_ was defined as the absorbance of control at 734 nm, and *A*_1_ and *A*_2_ were defined as the absorbance of the sample without the ABTS^+^ solution and with added ABTS^+^ solution, respectively.

### 2.9. Experimental Design

#### Response Surface Optimization Experiment

Based on the single-factor experimental design (data not shown), a four-factor and three-level Box–Behnken design (BBD) with response surface methodology (RSM) was employed in this study to determine the optimal combination of the independent variables. The flavonoid yield (y, mg/g) was considered as the dependent variable. The selection and range of these factors were based on our preliminary single-factor experimental data. For a BBD with four independent variables at three levels, 29 experimental runs were required. The design of the experiments is given in Table 1. 

The relationship between the dependent and independent variables was explained by a second-order polynomial model, as follows:

(4) $$Y=\beta_{0}+\sum_{i=1}^{k}\beta_{i}X_{i}+\sum_{i=1}^{k}\beta_{ii}X_{i}^{2}+\sum_{i=1}^{k-1}\sum_{j>1}^{k}\beta_{ij}X_{i}X_{j}$$

where *Y* is the response variable, X*_i_* and X*_j_* are independent variables, and *k* is the number of tested variables (*k* = 4). The regression coefficient is defined as *β*_0_ for the intercept and *β_i_* for linear terms, *β_ii_* for quadratic terms and *β_ij_* for cross-product terms.

### 2.10. Statistical Analysis

All statistical analyses were performed using SPSS 22.0 (SPSS Inc., Chicago, IL, USA). Results were expressed as the mean ± SD (*n* = 3) for each analysis. Differences were estimated by analysis of variance (ANOVA) followed by LSD test and differences were designated as statistically significant when *p* < 0.05. Correlation analysis was performed to examine relationships between antioxidant activities and individual flavonoids. The regression analysis and the optimization of RSM were analyzed using Design Expert 8.0.5 software (Stat-Ease Inc., Minneapolis, MN, USA). An analysis of variance (ANOVA) was carried out to assess the statistical significance (*p* < 0.05) of independent variables. The lack of fit, coefficient of determination (R^2^), adjusted coefficient of determination (adj. R^2^), coefficient of variation (C.V.) and F-values were used to evaluate the adequacy of the models. 

## 3. Results and Analysis

### 3.1. Optimization of the UHPE Procedure 

#### 3.1.1. Fitting the Model

The experimental conditions and results of 29 runs are presented in Table 2. The results of the ANOVA are shown in Table 3. 

The model’s F-value of 276.25 implies the model is very significant (*p* < 0.001). There is only a 0.01% chance that a model’s F-Value this large could occur due to noise. Values of “Prob > F” less than 0.0500 indicate that model terms are significant. In this case X_1_, X_2_, X_3_, X_4_, X_1_X_2_, X_3_X_4_, X_1_^2^, X_2_^2^, X_3_^2^, X_4_^2^ are significant model terms. 

The lack of fit of each model was not significant (*p* >0.05), indicating that the developed model adequately explains the relationship between the independent variables and responses. The lack of fit F-value of 1.77 implies that the lack of fit is not significant relative to the pure error. The predicted R^2^ of 0.9820 is in reasonable agreement with the adjusted R^2^ of 0.9928, which indicated a high degree of correlation between the experimental and predicted values. The adequate precision measures the signal-to-noise ratio, where a value greater than 4 is desirable. The ratio of 53.151 indicates an adequate signal. This model can be used to navigate the design space.

**Figure 1:**
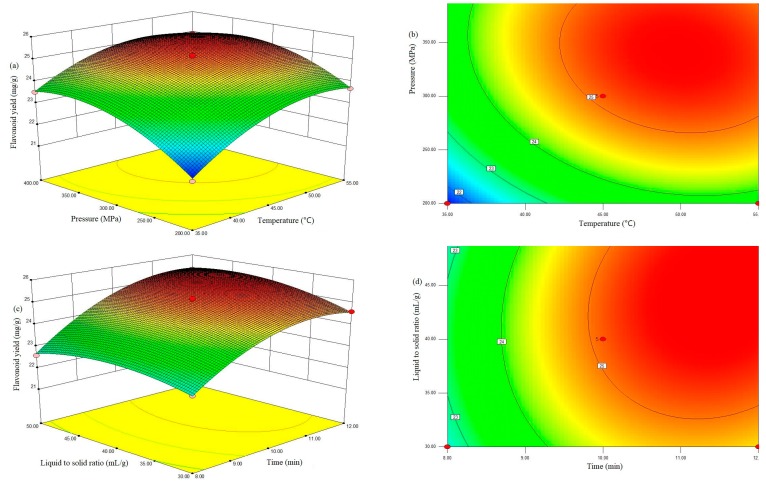
Response surface plots (**a**,**c**) and contour plots (**b**,**d**) of flavonoid yield affected by extraction temperature (*X*_1_), extraction pressure (*X*_2_), extraction time (*X*_3_) and liquid-to-solid ratio (*X*_4_).

The three-dimensional (3D) response surface plot can provide a visual interaction between two parameters and facilitate the optimal conditions for maximal response data [3]. For instance, in Figure 1a, the TFC increased with increasing pressure and temperature as time and the co-solvent-to-solid ratio were kept constant at 10.0 min and 40 mL/g, respectively. Moreover, the interaction effects of any other extraction variables on the TFC were similar (Figure 1b–d, respectively). The pressure had a greater effect because of its significant effects on viscosity, the surface tension of solvent, and the diffusion and solubility of the target compounds in solvents. Nevertheless, an excessively high pressure can damage the target compounds and lead to a slight decreasing in the flavonoid yield [24].

Moreover, the interaction effects of any other extraction variables on the TFC were similar (Figure 1b–d, respectively). 

By applying a multiple regression analysis, the relationship between the tested independent variables and the response is explained in Equation (5), in which X_i_ is a real value.

Y = 25.1 + 0.86 X_1_ + 0.77 X_2_ + 1.14 X_3_ + 0.21 X_4_ − 0.21 X_1_ X_2_ + 0.058 X_1_ X_3_ + 0.045 X_1_ X_4_ + 0.09 X_2_ X_3_ − 0.058 X_2_ X_4_ + 0.19 X_3_ X_4_ − 0.82 X_1_^2^ − 0.84 X_2_^2^ − 0.82 X_3_^2^ − 0.45 X_4_^2^ (5)

#### 3.1.2. Model Validation 

The optimal conditions for flavonoid extraction were as follows: a temperature of 50.0 °C, pressure of 342.39 MPa, time of 11.56 min, and liquid-to-solid ratio of 43.95 mL/g. To examine the validity of the model, an extraction was performed with five replicates under these conditions. The measured values (25.45 ± 0.21 mg/g) lay within a 95% mean confidence interval of the predicted value (25.9656 mg/g). These results confirmed the predictability of the model. 

### 3.2. Antioxidant Activity

**Figure 2:**
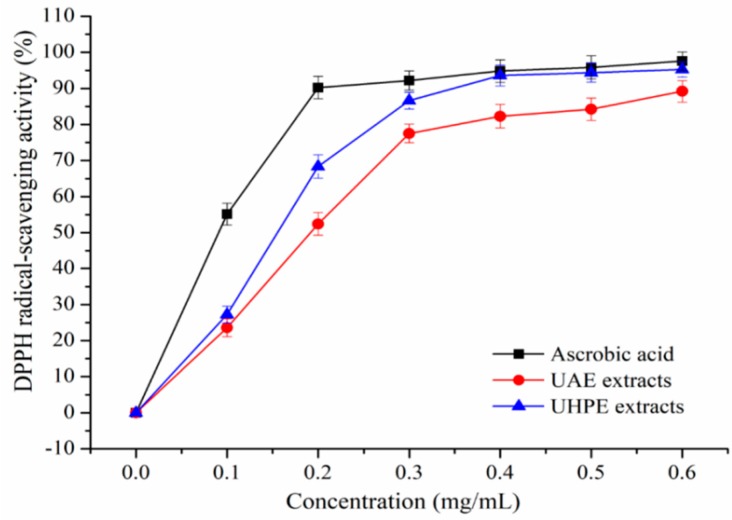
2,2-Diphenyl-1-picrylhydrazyl (DPPH) radical-scavenging activity of ultrasound-assisted extraction (UAE) and ultra-high pressure extraction (UHPE) extracts.

**Figure 3:**
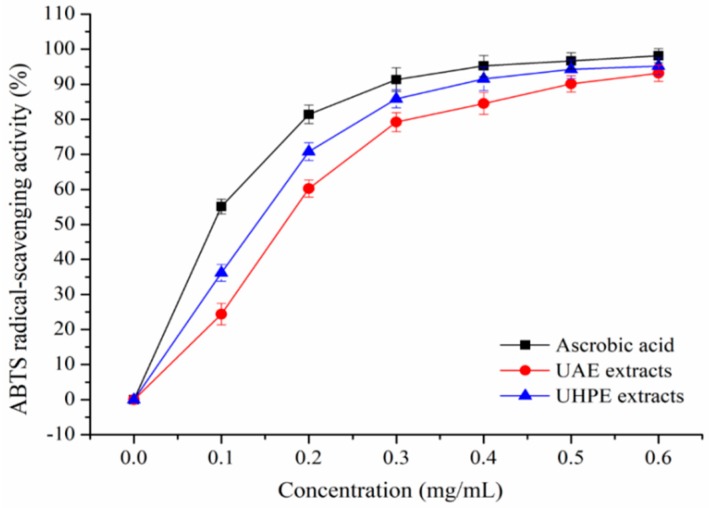
2,2′-Azino-bis(3-ethylbenzothiazoline-6-sulphonic acid) (ABTS) radical-scavenging activity of UAE and UHPE extracts.

The DPPH and ABTS^+^ scavenging activity of XJL extracts under optimum conditions were shown in Figure 2 and Figure 3. Extraction methods affect the antioxidant activities of plant extracts [25].

It was observed that XJL extracts exhibited notable DPPH and ABTS radical-scavenging activities in a concentration-dependent manner. When the concentration of the flavonoids increased from 0.1 to 0.6 mg/mL, the DPPH radical-scavenging activity of the UHPE extracts increased from (27.22 ± 2.36) % to 95.23 ± 2.11%, and the DPPH radical-scavenging activity of UAE extracts increased from 23.49 ± 2.33% to (89.02 ± 2.51) %. Additionally, at a concentration of 0.4 mg/mL, the scavenging activity of UHPE extracts on DPPH increased to (93.55 ± 2.86) %, approaching that of ascorbic acid at (94.79 ± 3.13)% and higher than that of UAE extracts at (82.34 ± 2.88) %. As shown in Figure 3, at a concentration of 0.4 mg/mL, the ABTS radical-scavenging activities of ascorbic acid, UAE and UHPE extracts were (95.22 ± 2.98) %, (84.47 ± 3.02) % and (91.55 ± 3.31) %, respectively.

At all measured concentrations, the UHPE extracts showed higher DPPH and ABTS radical-scavenging activities than UAE extracts, although they were a little lower than ascorbic acid. This may be due to fact that ultra-high pressure processing can enhance the mass transfer rate and increase the extraction rate of bioactive compounds [26,27].

### 3.3. HPLC Analyses of UAE and UHPE Extracts

**Figure 4:**
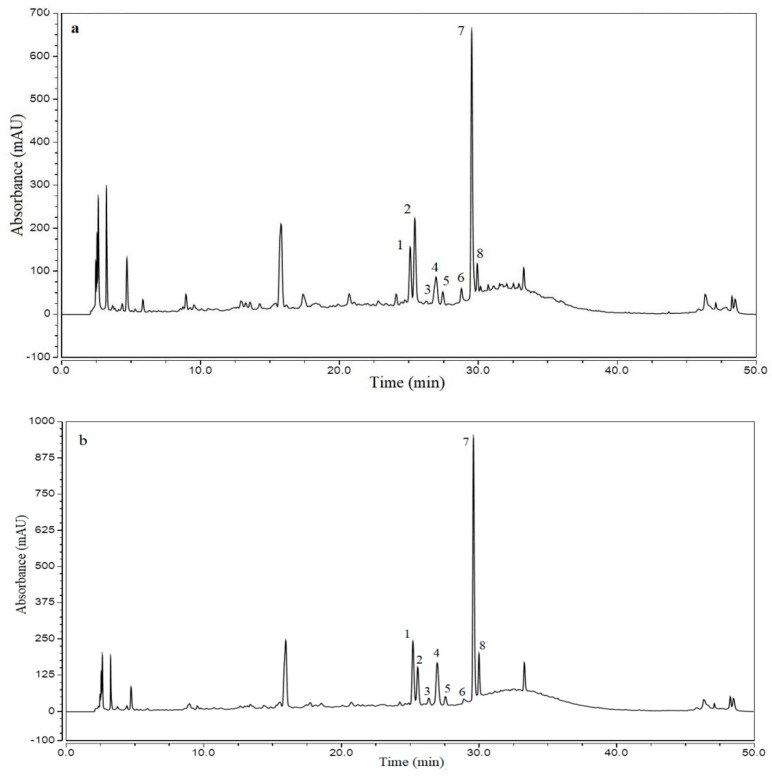
HPLC chromatograms of flavonoids in the XJL extracts at 280 nm. The peak numbering corresponds to the compounds in Table 4. (**a**): UAE extract, (**b**): UHPE extract.

The UAE and UHPE extracts (obtained under optimum conditions) were analyzed using HPLC and UPLC-ESI-MS. The HPLC chromatogram of the flavonoids in various extracts is shown in Figure 4. The compound characteristics, identities and quantifications are presented in Table 4.

As shown in Table 4, eight individual flavonoids were tentatively identified and quantified. The chromatograms of the extracts obtained by the two extraction methods used in this study were found to be quite similar, and all of the flavonoids were kaempferol and quercetin glycosides. There are two flavonols as well as quercetin-3-*O*-arabinosyl-rhamnoside and quercetin-3-*O*-xylosyl-rhamnoside which were not commonly found in plants. A previous study showed that in wild jujube leaf tea samples, contents of these two compounds were distributed at 1.6–4.4 and 0.2–0.56 mg/g, respectively [3]. In our study, the contents of quercetin-3-*O*-arabinosyl-rhamnoside in UAE and UHPE extracts were 10.180 ± 0.05 and 13.912 ± 0.070 mg/g, respectively. The contents of quercetin-3-*O*-xylosyl-rhamnoside in UAE and UHPE extracts were 1.290 ± 0.022 and 2.052 ± 0.027 mg/g, respectively. These two compounds with special sugar moiety may have special bioactivity for human health; however, this needs to be further evaluated.

There were significant differences (*p* < 0.05) in the contents of the seven flavonoids, excepting kaempferol 3-*O*-robinobioside. The total content of flavonoids extracted by UHPE was significantly higher (*p* < 0.05) than UAE.

The results were similar to that found during the UHPE of flavonoids, phenolic acids, polysaccharides, anthocyanins and sulforaphane from Chilean papaya seeds [28], propolis [29] and grape skin [30], respectively.

Presumably, these elevated levels can be attributed to many factors. UHPE can not only destroy the cell wall and cell membrane of plant materials, but also cause the deprotonation of charged groups and the destruction of salt bridges and hydrophobic bonds, rendering the flavonoids more available to extraction up to equilibrium [20]. According to the mass transfer and phase behavior theory, the higher the pressure, the more solvent can enter into the cell and the more compounds can permeate the cell membrane, leading to rapid permeation. Consequently, the flavonoid concentration between the cell interior and the exterior of cell membranes can reach equilibrium in a short time [31]. UHPE also has the ability to reduce the solvent pH, which might also enhance bioactive compound extraction because most of the compounds are more stable at low pH values [27].

Pharmacological and nutritional studies have found that flavonoids exhibit various biochemical activities, such as antioxidant, immunoregulatory and anticancer effects [3,4,5,6]. Therefore, these eight compounds in the extracts may be responsible for the antioxidant activity observed in flavonoids obtained by UAE and UHPE. XJL may be an excellent functional food material for preventing relevant disease.

### 3.4. Correlation Analysis

The correlations between antioxidant activities, TFC and the contents of individual flavonoids were analyzed (Table 5) to establish which compounds or family of compounds contributed to each antioxidant activity assay.

For DPPH assay, highly positive significant correlations (*r* = 0.900–0.996, *p* < 0.01) were observed for ABTS, TFC, Qa, Ql and Qx. For ABTS assay, highly positive significant correlations (*r* = 0.903–0.996, *p* < 0.01) were found for DPPH, TFC, Qa and Ql. TFC values were well positively correlated with DPPH, ABTS, Qr, Qa, Kr, Ql and Qx (*r* > 0.900, *p* < 0.01).

These observations are consistent with published data. It has been strongly substantiated that flavonoids are powerful radical quenchers in various systems. This phenomenon is presumably attributed to the high scavenging power of flavonoids, making it capable of rapidly deactivating a wide variety of radicals via electron transfer [32,33].

However, for both DPPH, ABTS assay and TFC, low correlations were all observed for R, Qu and A (*r* = 0.554–0.779, *p* < 0.05). These differences observed between assays are related to the varieties, contents, molecular structures, and physical properties of individual compounds [32,34].

Thus, stereoisomerism, functional group distribution and any other structural parameters such as the oxidation state of the C-ring and the hydroxylation and methylation patterns are also expected to affect the final value [32,33,34,35]. It could be speculated that individual flavonoids exert their antioxidant activity differently. They might act individually, synergistically or antagonistically [35]. The structure–activity relationship and molecular mechanisms need to be clarified with further studies.

## 4. Conclusions

In this study, the extraction of flavonoids from XJL by UHPE was optimized by RSM, and the antioxidant activities and the flavonoid profiles of the extracts were evaluated compared to those of UAE. The optimal conditions to obtain the highest flavonoid yield (25.45 ± 0.21 mg/g) of XJL were determined to be 50.0 °C, 342.39 MPa, and 11.56 min, with a 43.95 mL/g liquid-to-solid ratio. Compared to UAE, UHPE provided significant increases in flavonoid yield and antioxidant activity. Eight flavonoids were tentatively identified and quantified as kaempferol and quercetin glycosides, including quercetin 3-*O*-robinobioside, rutin (quercetin 3-*O*-rutinoside), hyperoside (quercetin-3-*O*-d-galactoside), quercetin-3-*O*-d-glucoside, kaempferol 3-*O*-robinobioside, kaempferol-3-*O*-glucoside, quercetin-3-*O*-β-l-arabinosyl-(1→2)-α-l-rhamnoside and quercetin-3-*O*-β-d-xylosyl-(1→2)-α-l-rhamnoside. Quercetin-3-*O*-β-l-arabinosyl-(1→2)-α-l-rhamnoside was found to have the highest content in the two extracts. Based on the results, we concluded that UHPE is a promising technique for extracting flavonoids from XJL, which might play an important role in improving the extraction yield of antioxidant flavonoids and sustainably using XJL resources.

## Figures and Tables

**Table 1:** Levels and codes of variables chosen for the design.

Code	X_1_: Temperature (°C)	X_2_: Pressure (MPa)	X_3_: Time (min)	X_4_: Liquid-to-Solid Ratio (mL/g)
−1	35	200	8	30:1
0	45	300	10	40:1
+1	55	400	12	50:1

**Table 2:** Box–Behnken design and response values for the flavonoid yield of Xinjiang jujube leaves (XJL).

Runs	X_1_: Temperature (°C)	X_2_: Pressure (MPa)	X_3_: Time (min)	X_4_: Liquid-to-Solid Ratio (*V*:*m*)	Y: Flavonoid Yield (mg/g)
1	45	300	10	40	25.14
2	55	200	10	40	23.69
3	55	300	10	50	25.04
4	45	200	12	40	23.83
5	35	300	10	50	23.19
6	55	300	10	30	24.43
7	45	300	10	40	25.03
8	45	300	10	40	24.99
9	55	400	10	40	24.91
10	55	300	12	40	25.44
11	55	300	8	40	23.05
12	45	300	12	30	24.59
13	45	200	8	40	21.74
14	35	300	10	30	22.76
15	45	400	10	50	24.78
16	35	400	10	40	23.52
17	35	300	8	40	21.59
18	45	300	10	40	25.14
19	45	300	10	40	25.19
20	45	200	10	50	23.26
21	45	400	8	40	22.92
22	45	300	8	50	22.59
23	45	400	10	30	24.48
24	45	300	12	50	25.29
25	45	300	8	30	22.66
26	35	300	12	40	23.75
27	35	200	10	40	21.46
28	45	200	10	30	22.73
29	45	400	12	40	25.37

**Table 3:** Analysis of variance for the response surface model for the flavonoid yield of XJL.

Source	Sum of Squares	df	Mean Square	F Value	*p*-Value Prob > F
Model	42.38	14	3.03	276.25	<0.0001
X_1_: temperature	8.82	1	8.82	805.18	<0.0001
X_2_: pressure	7.16	1	7.16	653.46	<0.0001
X_3_: time	15.69	1	15.69	1431.42	<0.0001
X_4_: Liquid–material ratio	0.52	1	0.52	47.53	<0.0001
X_1_X_2_	0.18	1	0.18	16.1	0.0013
X_1_X_3_	0.013	1	0.013	1.21	0.2905
X_1_X_4_	8.10 × 10^−3^	1	8.10 × 10^−3^	0.74	0.4044
X_2_X_3_	0.032	1	0.032	2.96	0.1076
X_2_X_4_	0.013	1	0.013	1.21	0.2905
X_3_X_4_	0.15	1	0.15	13.53	0.0025
X_1_^2^	4.4	1	4.4	401.08	<0.0001
X_2_^2^	4.58	1	4.58	418.31	<0.0001
X_3_^2^	4.41	1	4.41	402.29	<0.0001
X_4_^2^	1.32	1	1.32	120.88	<0.0001
Residual	0.15	14	0.011		
Lack of fit	0.13	10	0.013	1.77	0.3062
Pure error	0.028	4	7.07 × 10^−3^		
Cor total	42.54	28			
R^2^	0.9964				
Adj R^2^	0.9928				
Pred R^2^	0.9820				
Adeq precision	53.151				

**Table 4:** Flavonoid compositions in the extracts identified and quantified by HPLC and UPLC-ESI-MS.

Peak	Rt1 ^a^	Rt2 ^a^	Compounds	Calculated MW	[M + H]+/[M − H]− b	Δ mmu	Fragment Ions b	MS^2^	Quantification (mg/g)		Ref.
	(Min)	(Min)		(Da)			(*m*/*z*)	(*m*/*z*)	UAE Extract	UHPE Extract	
1	7.71	23.93	Quercetin-3-*O*-robinobioside	610.1534	611.1599	−1.230	303.0495; 465.1025	303.0496; 465.1023	1.820 b ± 0.120	2.987 a ± 0.017	[4]
2	7.94	24.38	Rutin (Quercetin-3-*O*-rutinoside)	610.1534	611.1605	−0.680	303.0498; 465.1028	303.0496; 465.1023	2.740 a ± 0.026	1.631 b ± 0.021	[4]
3	8.11	24.98	Hyperoside(Quercetin-3-*O*-β-d-galactoside)	464.0955	465.1026	0.118	303.0460	303.0495	0.200 b ± 0.011	0.419 a ± 0.010	[4]
4	8.33	25.32	Quercetin-3-*O*-β-d-glucoside	464.0955	465.1026	0.118	303.0495	303.0495	0.710 b ± 0.021	1.208 a ± 0.025	[4]
5	8.89	25.62	Kaempferol-3-*O*-robinobioside	594.1585	595.1660	0.203	287.0549; 449.1080	287.0548; 449.1084	0.360 a ± 0.014	0.403 a ± 0.016	[4]
6	9.96	28.23	Kaempferol-3-*O*-glucoside	448.1006	449.1109	0.162	287.0602	287.0548	00.390 a ± 0.022	0.232 b ± 0.030	[4]
7	10.11	28.73	Quercetin-3-*O*-β-l-arabinosyl-(1→2)-α-l-rhamnoside	580.1428	581.1503	0.054	303.0469; 449.1109	303.0499; 449.1081	10.180 b ± 0.050	13.912 a ± 0.070	[4]
8	10.42	29.25	Quercetin-3-*O*-β-d-xylosyl-(1→2)-α-l-rhamnoside	580.1428	581.1505	0.534	303.0500; 449.1082	303.0499; 449.1081	1.290 b ± 0.022	2.052 a ± 0.027	[4]
Total									17.690 b ± 0.286	22.844 a ± 0.226	[4]

* The results are presented as the mean ± SD of three replicate analyses. Different letters in the same row indicate significant differences (*p* < 0.05) between the samples. a Rt1 was obtained in the UPLC-ESI-MS analysis, and Rt2 was obtained in the HPLC-UV analysis. b Both positive and negative ion modes were used in the UPLC–ESI-MS analysis; fragment ions for phenolic acids were obtained in negative mode, and fragment ions for others were obtained in positive mode.

**Table 5:** Correlation (*r* values) between the antioxidant activity, total flavonoid content (TFC) and individual flavonoids.

	DPPH	ABTS	TFC	Qr	R	Qa	Qu	Kr	A	Ql	Qx
DPPH	-	0.996 **	0.909 **	0.888 **	0.637 *	0.908 **	0.617 *	0.842 **	0.609 *	0.915 **	0.907 **
ABTS	0.996 **	-	0.909 **	0.894 **	0.666 **	0.904 **	0.579 *	0.855 **	0.639 *	0.910 **	0.896 **
TFC	0.909 **	0.909 **	-	0.993 **	0.779 **	0.990 **	0.583 *	0.962 **	0.745 **	0.992 **	0.971 **

Abbreviation: Qr: quercetin3-*O*-robinobioside, R: rutin, Qa: quercetin-3-*O*-β-d-galactoside, Qu: quercetin-3-*O*-β-d-glucoside, Kr: kaempferol 3-*O*-robinobioside, A: Astragalin (Kaempferol-3-*O*-glucoside), Ql: quercetin-3-*O*-β-l-arabinosyl-(1→2)-α-l-rhamnoside, Qx: quercetin-3-*O*-β-d-xylosyl-(1→2)-α-l-rhamnoside. DPPH, ABTS: radical scavenging activity, TFC: total flavonoid content. Values of r > |−0.900| are in bold. * Significant at *p* < 0.05. ** Significant at *p* < 0.01).
